# Supplementary material for: The impact of environmental regulatory instruments on agribusiness technology innovation—A study of configuration effects based on fsQCA
Source: PLoS One. 2024 Jan 18;19(1):e0294662. doi: 10.1371/journal.pone.0294662 (PMC10796004; doi:10.1371/journal.pone.0294662)
Supplement: Attachment — Submitted filename: renamed_ee900.docx [file pone.0294662.s002.docx]

Dear author,

After carefully examining your manuscript, we believe that this is a well-written paper containing interesting results that merit publication. For the benefit of the readers, however, a number of points need clarifying and certain statements require further justification. They are given as below:

1. Introduction: Agriculture-related businesses need to be more closely connected. It is essential to concentrate more on agricultural businesses than on other enterprises, taking into account the analysis of the reported outcomes.
2. Supporting Literature: The majority of the research discussed in this article relates to China, and it is advised that it be better applied in conjunction with pertinent international research.
3. Limitations and Further Research: It would be helpful if you discussed the limitations of your study more explicitly. The manuscript lacks directions on future research or the next steps following this study’s completion. Please consider providing some guidance in this area.

In conclusion, we think the subject matter covered in this paper is significant, the research techniques employed are innovative, and the article can be considered publishable after appropriate minor revisions. Look forward to reading your essay once it has been revised.

Best regards,
